# Ultrahigh Carrier Mobility in Two-Dimensional IV–VI Semiconductors for Photocatalytic Water Splitting

**DOI:** 10.3390/molecules28104126

**Published:** 2023-05-16

**Authors:** Zhaoming Huang, Kai Ren, Ruxin Zheng, Liangmo Wang, Li Wang

**Affiliations:** 1 School of Mechanical Engineering, Nanjing University of Science and Technology, Nanjing 210094, China; 2 School of Mechanical and Electronic Engineering, Nanjing Forestry University, Nanjing 211189, China; kairen@njfu.edu.cn (K.R.);; 3 School of Mechanical Engineering, Wanjiang University of Technology, Ma’anshan 243031, China; 4 School of Mechanical Engineering, Southeast University, Nanjing 211189, China; 5 Office of Academic Affairs, Xuancheng Vocational and Technical College, Xuancheng 242000, China

**Keywords:** IV–VI monolayers, mechanical property, carrier mobility, hydrogen evolution reaction

## Abstract

Two-dimensional materials have been developed as novel photovoltaic and photocatalytic devices because of their excellent properties. In this work, four δ-IV–VI monolayers, GeS, GeSe, SiS and SiSe, are investigated as semiconductors with desirable bandgaps using the first-principles method. These δ-IV–VI monolayers exhibit exceptional toughness; in particular, the yield strength of the GeSe monolayer has no obvious deterioration at 30% strain. Interestingly, the GeSe monolayer also possesses ultrahigh electron mobility along the *x* direction of approximately 32,507 cm^2^·V^−1^·s^−1^, which is much higher than that of the other δ-IV–VI monolayers. Moreover, the calculated capacity for hydrogen evolution reaction of these δ-IV–VI monolayers further implies their potential for applications in photovoltaic and nano-devices.

## 1. Introduction

Since graphene was proposed as a representative of two-dimensional (2D) materials in 2004 [1], considerable efforts have been made to explore its excellent mechanical [2], electronic [3], magnetic [4] and chemical [5] properties, showing promise for its widespread use in nano-devices. Since then, other 2D materials have also been studied by researchers [4,6,7,8,9,10]. Among them, transition metal dichalcogenides (TMDs) have also attracted great interest due to their novel properties [11,12], such as WSe_2_, MoSSe and MoS_2_. Monolayered WSe_2_ possesses outstanding mechanical properties, and experiments have shown that when the layers of the WSe_2_ is increased to 2–4, the photoluminescence (PL) is significantly enhanced by 2% under tensile strain [13]. It is reported that the carrier mobility of the MoS_2_ monolayer is equivalent to that of its nanobelts. In addition, the carrier mobility in MoS_2_ nanobelts can be made more robust while reducing the size of the monolayered MoS_2_ [14]. The TMD materials with a Janus structure further result in an excellent performance [15]. MoSSe shows unusual properties in adsorption, with high gas sensitivity and surface and strain selectivity [16]. In order to expand the applications of 2D materials, the stacking of two different materials into a heterostructure using van der Waals (vdWs) force is a popular method. Based on this, different properties of materials can be induced at the interface. The Janus TMD vdW heterostructure also shows promise for photocatalytic and thermal applications [17,18,19].

The carrier mobility of 2D materials is a critical property used in the applications of nano-devices [20]. The carrier mobility of the CaP_3_ monolayer is calculated to be 19,930 cm^2^·v^−1^·s^−1^, while the carrier mobility can be increased to be 22,380 cm^2^·v^−1^·s^−1^ by connecting two layers of CaP_3_ together using vdWs forces [21]. A family of Li*_x_*B*_y_* monolayers were investigated using the evolutionary structure search method, and the monolayered Li_2_B_6_ showed a high hole mobility of approximately 6.8 × 10^3^ cm^2^·v^−1^·s^−1^, showing that it can be used in high-speed electronic devices [22]. The high carrier mobility also contributes to the efficient photocatalytic properties in the hydrogen evolution reaction (HER). The ability of the HER is determined by the interaction between the intermediate and photocatalyst, which is evaluated using Gibbs free energy [23,24]. Importantly, the HER also can be tuned via defect [25,26], nanonization [27], heterostructures [28], etc. Moreover, the suitable band energy of the semiconductor is also critical in order to decompose the water so that the conduction band minimum (CBM) is more positive than −4.44 eV for the redox potentials (H^+^/H_2_) and the valence band maximum (VBM) is more negative than −5.67 eV for the oxidation potential (O_2_/H_2_O) at pH 0 [29]. More recently, the δ-IV–VI monolayers, GeS, GeSe, SiS and SiSe, were predicted to possess an excellent light absorption performance (even at up to 7.8 × 10^5^ cm^−1^ for SiSe), meaning that they can be considered as promising photocatalysts. However, to be candidates for water splitting, the carrier mobility and the HER properties need to be further developed.

In this work, the density functional theory (DFT) is applied to systematically investigate the mechanical and electronic properties of the δ-IV–VI monolayers (GeS, GeSe, SiS and SiSe). Furthermore, the electronic and stress–strain responses are addressed. Next, the carrier mobility and the hydrogen evolution reaction of the δ-GeS, δ-GeS, δ-SiS and δ-SiSe monolayers are explored.

## 2. Results and Discussion

**Figure 1:**
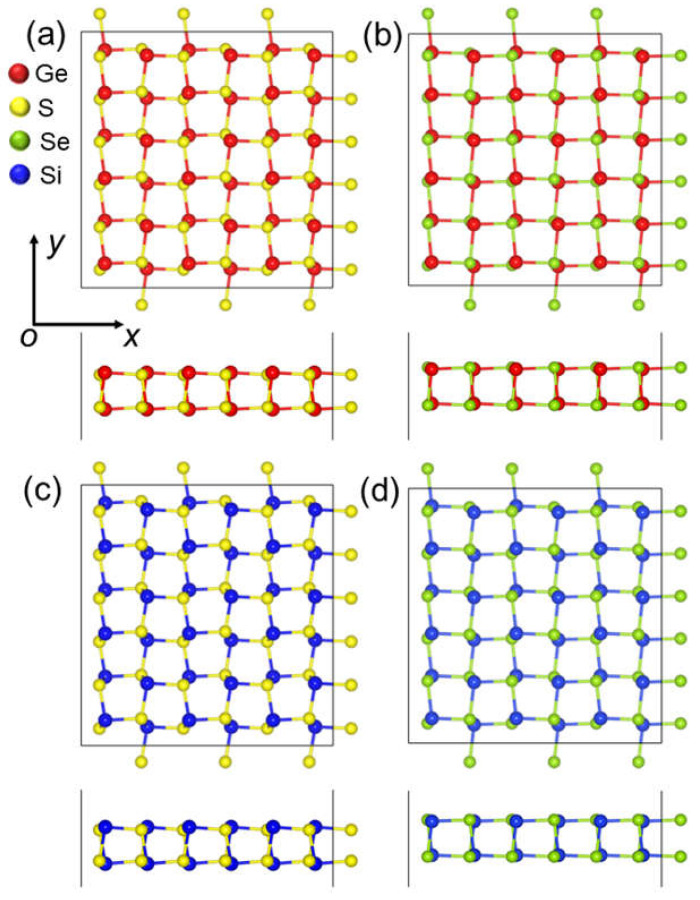
The top and side views of the atomic structures of the (**a**) GeS, (**b**) GeSe, (**c**) SiS and (**d**) SiSe monolayers.

First, the structure of the δ-GeS, δ-GeS, δ-SiS and δ-SiSe monolayers were optimized as shown in Figure 1. The lattice parameters in the *x* (or *y*) direction were calculated as 5.58 Å (or 5.76 Å), 5.83 Å (or 5.81 Å), 5.50 Å (or 5.67 Å) and 5.69 Å (or 5.73 Å), respectively, for the δ-GeS, δ-GeS, δ-SiS and δ-SiSe monolayers. Moreover, the bond lengths between the Ge–S, Ge–S, Si–S and Si–Se monolayers were obtained as 2.42, 2.54, 2.32 and 2.44 Å, which are in good agreement with previous research [30]. These IV–VI monolayers possess a space group of *Pca*2_1_, which was also reported in a previous investigation [30]. The cohesive energy (*E*_co_) of the δ-IV–VI monolayers was calculated using *E*_co_ = (4*E*_X_ + 4*E*_Y_ − *E*_XY_)/8, where *E*_X_, *E*_Y_ and *E*_XY_ are the total energies of a Ge (or Si) atom, a Se (or S) atom and the δ-IV–VI monolayer, respectively. The obtained cohesive energy of the GeS, GeSe, SiS and SiSe monolayers are 3.61, 3.37, 3.81 and 3.51 eV/atom, which are comparable with the values of phosphorene (approximately 3.48 eV/atom), germanene (approximately 3.24 eV/atom) and silicene (approximately 3.91 eV/atom) [31]. The obtained cohesive energy of SiS is larger than that of the puckered SiS (3.16 eV per atom) [32], and the obtained cohesive energy of the GeS and GeSe monolayers is also similar to that recently reported for GeS and GeSe with other phases [33,34,35]. Such IV–VI monolayers can be prepared in experiments using the chemical vapor deposition method and then isolated through mechanical, sonicated or liquid-phase exfoliation methods, which have been adopted to synthesize few-layer GaSe [36] and GeS [37].

**Figure 2:**
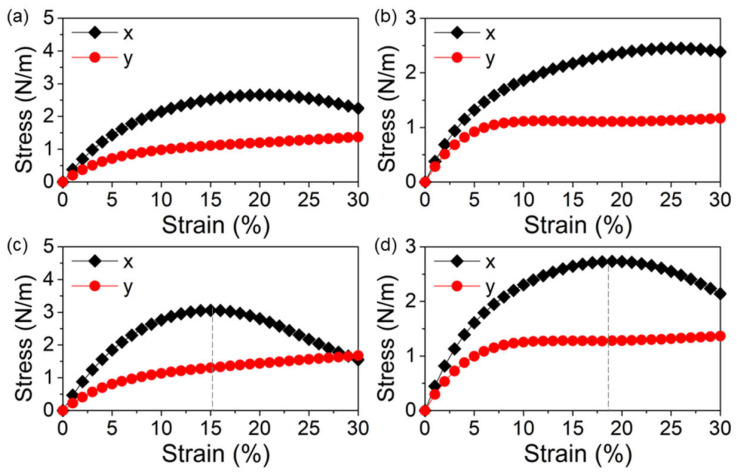
The strain–stress relationships of the (**a**) GeS, (**b**) GeSe, (**c**) SiS and (**d**) SiSe monolayers along the *x* and *y* directions.

The mechanical capacities of the δ-GeS, δ-GeS, δ-SiS and δ-SiSe monolayers were calculated by investigating the stress–strain response, as shown in Figure 2. The δ-GeS, δ-GeS, δ-SiS and δ-SiSe monolayers were more elastic in the *x* direction than the *y* direction. Through the linear fitting of the initial range (within 5%), the obtained Young’s moduli (*E*) of the δ-GeS, δ-GeS, δ-SiS and δ-SiSe monolayers were defined as *E* = ΔStress/ΔStrain, obtained to be approximately 34 N·m^−1^, 30 N·m^−1^, 39 N·m^−1^ and 28 N·m^−1^, respectively, along the *x* direction. Meanwhile, the Young’s moduli are calculated as 21 N·m^−1^, 15 N·m^−1^, 17 N·m^−1^ and 20 N·m^−1^, respectively, along the *y* direction for the δ-GeS, δ-GeS, δ-SiS and δ-SiSe monolayers, in accordance with our other report [30]. The SiS and SiSe monolayers have yield strengths of approximately 3.13 N·m^−1^ and 2.72 N·m^−1^ at the strains of 15% and 18% in the *x* direction, respectively, as shown by the gray dashed lines in Figure 2c,d.

**Figure 3:**
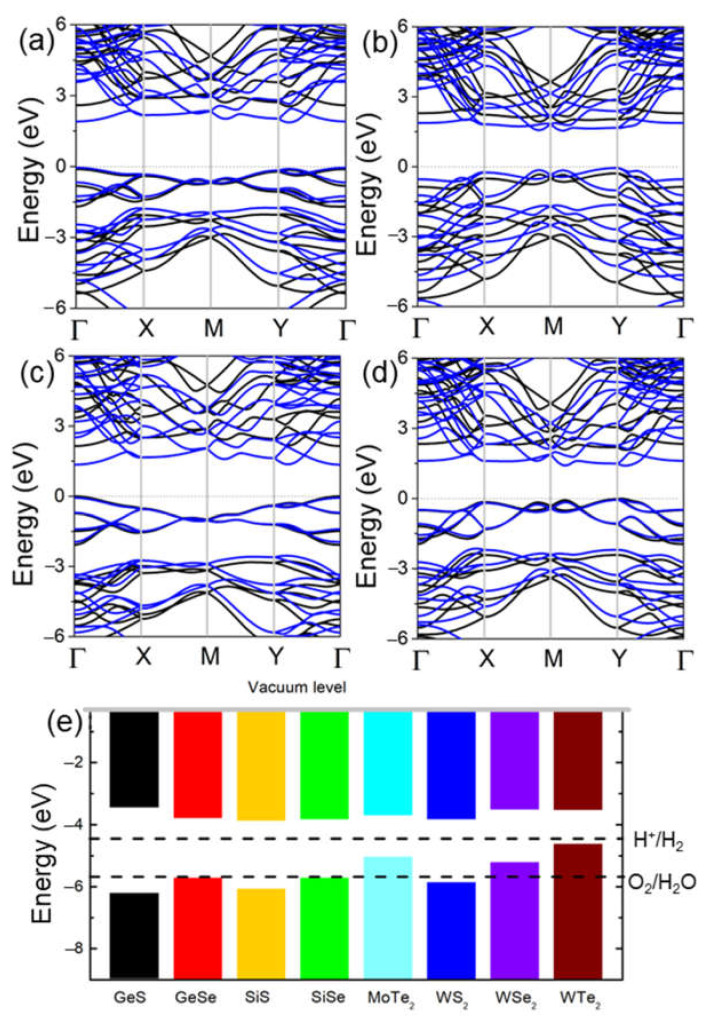
The DS-PAW calculated band structure of the (**a**) GeS, (**b**) GeSe, (**c**) SiS and (**d**) SiSe monolayers, (**e**) and the band alignment of these IV–VI monolayers compared with TMDs. The Fermi level is set as 0 eV. The blue and black lines represent the results of PBE and HSE06 calculations. The band edge energy was calculated with respect to the water oxidation (O_2_/H_2_O) and reduction (H^+^/H_2_) potentials at 0 pH.

The band structures of the δ-GeS, δ-GeS, δ-SiS and δ-SiSe monolayers shown in Figure 3 were determined using the PBE and HSE06 methods. One can see that all these monolayers are semiconductors, with bandgaps of approximately 2.65 eV (1.92 eV), 2.20 eV (1.60 eV), 2.15 eV (1.42 eV) and 2.08 eV (eV), being functional according to HSE06 (PBE). In Figure 3a–d, the δ-GeS, δ-GeS, δ-SiS and δ-SiSe monolayers possess almost exact bandgap structures, and the obtained bandgaps of these monolayers imply their decent application potential as photocatalysts for water splitting (larger than 1.23 eV) [38]. Furthermore, the band edge positions of these IV–VI monolayers are shown in Figure 3e at pH 0. Evidently, the δ-GeS, δ-GeS, δ-SiS and δ-SiSe monolayers demonstrate the sufficient energy of the CBM and VBM to induce the reductions and oxidations for water splitting. For comparative purposes, the band alignments of some TMD materials are also shown in Figure 3e, where one can see that only the WS_2_ monolayer has a suitable band energy for redox.

**Figure 4:**
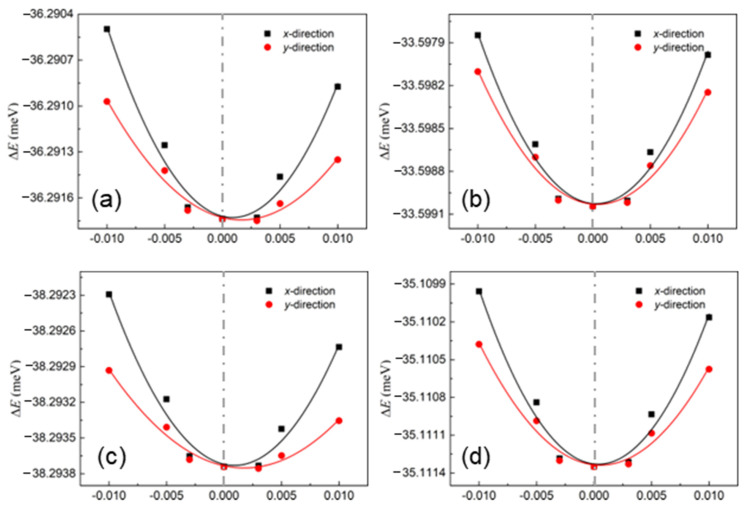
The energy differences among the (**a**) GeS, (**b**) GeSe, (**c**) SiS and (**d**) SiSe monolayers under different strains obtained by DS-PAW.

Next, the carrier mobility of the δ-GeS, δ-GeS, δ-SiS and δ-SiSe monolayers is investigated, considering their decent bandgaps. The effective masses (m^*^) of the electron and hole are determined by fitting the parabolic functions, which can be represented as:

(1) $$m^{*}=\pm \hbar^{2}{\left(\frac{d^{2}E_{k}}{\;dk^{2}}\right)}^{-1},$$

where *k* and *E_k_* are the wave vector and the corresponding electronic energy, respectively. Furthermore, the carrier mobility (*μ*) of these 2D materials is calculated using:

(2) $$\mu =\frac{e\hbar^{3}C}{k_{B}Tm^{*}m_{e}E_{d}{}^{2}},$$

where the temperature is explained by *T*, *e* is the electron charge, the Planck constant is determined by ħ, and *k*_B_ is the Boltzmann constant. The change in the band edge of these layered materials is evaluated using the deformation potential (*E*_d_). It is worth noting that the obtained deformation potentials are compared based on the vacuum level. Moreover, the elastic modulus is used with *C*, which is obtained using $C=\left[\partial^{2}E/\partial \varepsilon^{2}\right]/S$. Here, the total energy of the system is *E* and the area of the system is *S*. The energy differences among these δ-IV–VI monolayers are shown in Figure 4, and the fitted elastic moduli are summarized in Table 1.

**Figure 5:**
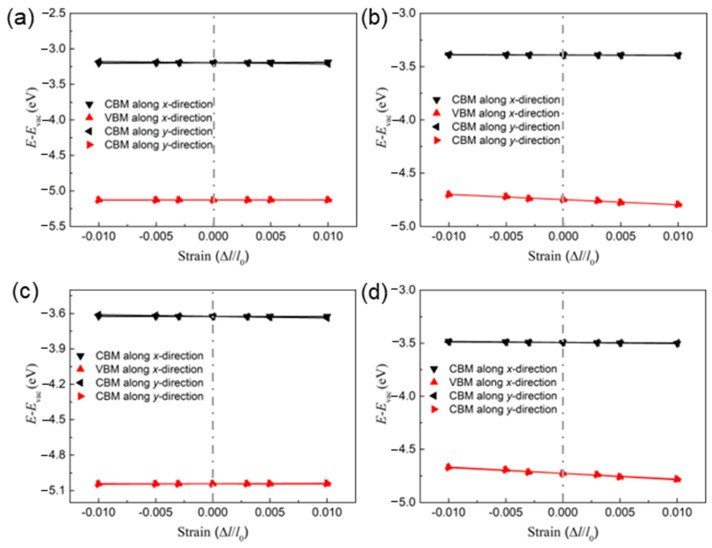
The changes in the band edge positions of the (**a**) GeS, (**b**) GeSe, (**c**) SiS and (**d**) SiSe monolayers under different strains obtained by DS-PAW.

As an important parameter of carrier mobility, the deformation potential is calculated using the strain response to the band edge positions. For this purpose, the ranges of external and uniaxial strain are controlled within 0.01. The changes in the band edge positions under different strains on the δ-IV–VI monolayers are exhibited in Figure 5. One can see that the energy of the VBM for the GeSe and SiSe monolayers is more sensitive than that of the others, which implies obstructed hole mobility. The Bardeen–Shockley deformation potential theory is considered in the calculations for the strain effect on the energy and the band edge position, which can be used to explore long-range electrostatic terms in the theory of electronic deformation potential, with the results also showing good agreement with the experiments [39]. As the effective masses calculated using the HSE06 functional may be inaccurate due to the effect of Hartree−Fock exchange [40], the PBE functional is used to predict the carrier mobility.

The carrier mobility of these δ-IV–VI monolayers was calculated, as shown in Table 1. Interestingly, the carrier mobility of the SiS monolayer in the *x* direction is approximately 10 times higher than that in the *y* direction, showing favorable carrier transport direction along *x* [41]. Meanwhile, the carrier mobility of the electrons is much higher than that of the holes in the GeSe and SiSe monolayers, which is advantageous for the separation of the photogenerated electrons and holes [42]. More importantly, the GeSe monolayer possess an ultrahigh electron mobility in the *x* direction of approximately 32,507 cm^2^·V^−1^·s^−1^, which is higher than that of black phosphorus [43]. In addition, the other obtained carrier mobilities of the GeS (465–1312 cm^2^·V^−1^·s^−1^), SiS (2202–2489 cm^2^·V^−1^·s^−1^) and SiSe (2997 cm^2^·V^−1^·s^−1^ for electron) are also higher than those of other novel 2D materials, such as WS_2_ (542 cm^2^·V^−1^·s^−1^) [44], MoS_2_ (201 cm^2^·V^−1^·s^−1^) [14], BSe (2396 cm^2^·V^−1^·s^−1^) [45], etc. Moreover, the obtained ultrahigh electron mobility of the GeSe monolayer in the *x* direction is attributed to its small deformation potential constant (about −0.92) and effective mass (approximately 0.11), suggesting the insensitivity of the band edge position to the external strain. Even though the GeS and SiS monolayers present small deformation potential constants, the carrier mobility is suppressed by the larger effective mass.

The catalytic properties of these IV−VI monolayers were also determined. The Gibbs free energy change (Δ*G*_H*_) of the GeS, GeSe, SiS and SiSe monolayers was investigated under standard conditions using:

Δ*G*_H*_ = Δ*E* + Δ*E*_zpe_ + *T*Δ*S*, (3)

where the total energy of the H-adsorbed IV−VI monolayers, the difference in the zero-point energies and the change in entropy caused by the adsorption are represented as Δ*E*, Δ*E*_zpe_ and Δ*S*, respectively. *T* is defined as 298.15 K. The active site is marked by the sign “*”. The HER characteristic is induced via two reactions:

∗ + H^+^ + e^−^ → H^∗^, (4)

H^∗^ + H^+^ + e^−^ → H_2_ + ∗. (5)

**Figure 6:**
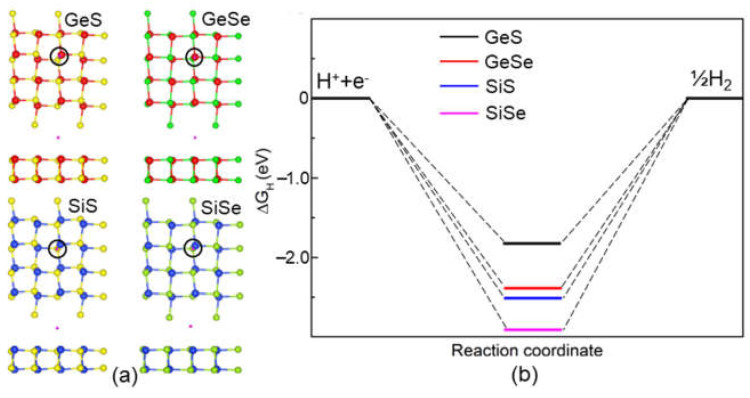
(**a**) The most favorable calculated adsorption sites and (**b**) the Gibbs free energies of the IV−VI monolayers obtained by DS-PAW.

Furthermore, the most favorable H-adsorbed sites of the systems are demonstrated in Figure 6a, and the calculated Gibbs free energies of these H-adsorbed GeS, GeSe, SiS and SiSe monolayers are obtained as −1.775 eV, 2.480 eV, 2.569 eV and 2.965 eV, respectively, as shown in Figure 6b. One can see that the GeS possesses an advantageous HER ability, which is even smaller than that of the MoSi_2_N_4_ (2.79 eV) and MoSi_2_N_4_ (2.51 eV) monolayers [46].

## 3. Computational Methods

In our simulations, the calculations for the structural optimization, electronic property, carrier mobility and the HER performances were calculated by Device Studio [Hongzhiwei Technology, Device Studio, Version 2021A, China, 2021. Available online: https://iresearch.net.cn/cloudSoftware] program, which provides a number of functions for performing visualization, modeling and simulation. And all that simulations using DS-PAW software are integrated in Device Studio program [47]. All the mechanical calculations were conducted based on DFT using the first-principles method with the Vienna ab initio simulation package (VASP) [48]. Generalized gradient approximation (GGA) was employed together with projector augmented wave potentials (PAW) to demonstrate core electrons [49,50]. The Perdew–Burke–Ernzerhof (PBE) functional was used to explain the exchange correlation functional. The DFT-D3 calculations were considered to describe the weak dispersion forces proposed by Grimme [39,51]. Furthermore, Heyd–Scuseria–Ernzerhof (HSE06) hybrid functional calculations were conducted to obtain more accurate electronic properties [52]. Monkhorst–Pack *k*-point grids of 11 × 11 × 1 and 17 × 17 × 1 in the first Brillouin zone (BZ) were used for the relaxation and self-consistent calculations, respectively. The spin–orbit coupling (SOC) effect is not considered in this work, because it has a negligible effect on the electron band structure of the studied materials. The energy cut-off was set as 550 eV. To avoid interaction between nearby layers, the vacuum space adopted was 20 Å. The convergence values for force and energy were set within 0.01 eV Å^−1^ and 0.01 meV, respectively.

## 4. Conclusions

In summary, the mechanical, electronic and HER properties of the δ-IV–VI monolayers, namely, GeS, GeSe, SiS and SiSe, were systematically investigated using first-principles calculations. The strain–stress relationships of these δ-IV–VI monolayers present a novel toughness along the *y* direction, while the yield strength was calculated for the SiS and SiSe monolayers in the *x* direction. The GeS, GeSe, SiS and SiSe monolayers showed semiconductor characteristics with a bandgap larger than 1.23 eV for water splitting. For this application, an excellent carrier mobility was determined for all these δ-IV–VI monolayers; in particular, the GeSe monolayer demonstrates an ultrahigh electron mobility in the *x* direction of approximately 32,507 cm^2^·V^−1^·s^−1^. Furthermore, the Gibbs free energies of these GeS, GeSe, SiS and SiSe monolayers were obtained and imply their potential for usage as photocatalysts for water splitting.

## Figures and Tables

**Table 1:** The obtained effective mass, elastic modulus, deformation potential constant and the carrier mobility of the hole (h) and the electron (e) for the GeS, GeSe, SiS and SiSe monolayers in the *x* and *y* directions using DFT calculations.

Materials	Direction	Carrier	*m*	*E* (eV)	*C*	*μ*
GeS	*x*	e^−^	2.95	0.79	41	465
		h^+^	−1.33	0.50		1246
	*y*	e^−^	0.16	−1.61	23	1140
		h^+^	−1.49	0.34		1312
GeSe	*x*	e^−^	0.11	−0.92	43	32,507
		h^+^	−1.32	5.10		45
	*y*	e^−^	0.35	−0.85	33	9543
		h^+^	−0.12	−4.82		439
SiS	*x*	e^−^	1.24	−0.53	50	2041
		h^+^	−1.04	−0.49		2489
	*y*	e^−^	0.82	−1.39	25	220
		h^+^	−1.33	0.74		411
SiSe	*x*	e^−^	2.70	−1.04	51	319
		h^+^	−1.28	−6.28		16
	*y*	e^−^	0.22	−0.97	34	2997
		h^+^	−0.66	−5.66		25

## Data Availability

The data presented in this study are available upon request from the corresponding author.

## Sample Availability

Samples of the compounds are available from the authors.
